# Sex-Based Differences in Polytraumatized Patients between 1995 and 2020: Experiences from a Level I Trauma Center

**DOI:** 10.3390/jcm13195998

**Published:** 2024-10-08

**Authors:** Valerie Weihs, Rita Babeluk, Lukas L. Negrin, Silke Aldrian, Stefan Hajdu

**Affiliations:** Department of Orthopedics and Trauma Surgery, Medical University of Vienna, 1090 Vienna, Austrialukas.negrin@meduniwien.ac.at (L.L.N.);

**Keywords:** polytrauma, epidemiology, sex differences, outcome

## Abstract

**Background/Objectives**: The aim of this study was to examine sex-related differences in the outcomes of polytraumatized patients admitted to a level I trauma center. **Methods**: This was a retrospective data analysis of 980 consecutive polytraumatized patients admitted to a single level I trauma center between January 1995 and December 2020. **Results**: Among all patients, about 30% were female, with a significantly higher age and significantly higher rates of suicidal attempts. No sex-related differences regarding injury severity or trauma mechanisms could be seen, but female patients had significantly higher overall in-hospital mortality rates compared to male patients. Even in the elderly group of patients, elderly female patients were significantly older compared to elderly male patients, with significantly increased lengths of hospital stay. In the elderly group of patients, no sex-related differences regarding injury severity, trauma mechanisms or mortality could be detected. Multivariate analysis revealed suicidal attempt, severe head injury and age > 54 years as independent prognostic factors in the survival of polytraumatized patients. **Conclusions**: Distinctive sex-related differences can be found, with female polytraumatized patients being significantly older and having higher overall mortality rates with significantly increased LOS. Our study suggests a strong sex-independent influence of age, suicidal attempt and severe head injury on the outcomes of polytraumatized patients.

## 1. Introduction

Multiple injuries to several body regions or organ systems with at least one injury being life-threatening (or the combination of several injuries) define the polytraumatized patient [1,2]. Traffic-related accidents, self-harm and interpersonal violence are still some of the most common causes of death and disability in adolescents and adults in industrialized countries, with increasing rates of falls leading to disability in the elderly population [3]. An important factor in the acute care of these patients is prognosis and risk prediction, which is influenced by different factors [4]. Previous studies have identified several conditions, such as the type and the site of injury and in-hospital complications, as risk factors in the elderly polytraumatized patient [5,6,7,8]. There are several risk adjustment models for the estimation of the risk of death in these patients [6,7,8,9,10,11]. The presence of severe traumatic brain injury (TBI) seems to have a significant influence, especially on the long-term outcomes in polytraumatized patients [12] with an increase in brain injury-related death rates documented over the last decades [13]. Little is known about the impact of sex-related factors on the outcomes of these patients. There are conflicting results from previous studies on whether sex has an impact on the outcomes of polytraumatized patients [14,15,16]. Higher age and longer in-hospital stay in female polytraumatized patients have recently been shown to suggest a higher risk for delayed long-term recovery [17]. Moreover, a beneficial effect of the female sex on younger, severely injured patients with lower in-hospitality mortality rates and decreased admissions to the intensive care unit (ICU) has been suggested [18]. These potential beneficial effects might derive from a possible protective role of sex-hormones in the younger female trauma patient [19].

To overcome these ongoing controversial debates, this large retrospective study was performed to determine a potential sex-related impact on the outcome of polytraumatized patients admitted to a level I trauma center.

## 2. Materials and Methods

In this study, 980 consecutive polytraumatized patients admitted to a level I trauma center from 1 January 1995 to 31 December 2020 were retrospectively included. Patients were included with severe injuries (ISS ≥ 18 points and an Abbreviated Injury Score (AIS) ≥ 3 points in at least 2 body regions [2]). Patients with isolated severe injuries, as well as patients with minor injuries (ISS < 18 points) and patients < 16 years of age, were excluded from this analysis. For the analysis of mortality, three time-dependent events were defined: acute-phase death (death within the first 24 h after the trauma), late-phase death (death after survival of the acute phase within the hospital stay) and overall death (death at any time within the hospital stay).

### 2.1. Patients’ Population

The patients’ data were extracted from a consecutive in-hospital database of polytraumatized patients. The hospital records of the patients were reviewed. Data analysis focused on baseline characteristics such as sex, age, injury severity and injury patterns, as well as in-hospital mortality. Severe traumatic brain injury (TBI) was defined as AIS > 3 points in the anatomical region. As possible prognostic factors, severe TBI, age, injury severity score (ISS), injury patterns and sex were defined. The follow-up period was counted from the date of the trauma to the date of the last known contact.

In light of previous studies, a cut-off value of 55 years of age was chosen with regard to the known independent negative influence of age ≥ 55 years on the survival of polytraumatized patients [12,20]. Additionally, this cut-off was defined to overcome a possible beneficial influence of sex hormones after trauma [19,21] assuming postmenopausal phases in women older than 54 years of age.

### 2.2. Statistical Analyses

Depending on the normal or skewed distribution of continuous variables, they are presented as means and standard deviations or medians and interquartile ranges. The Kolmogorov–Smirnov test was used to assess normal distribution. Categorical variables are presented with percentages. Descriptive analyses were used for demographic variables and clinical characteristics. The chi-square test was used for the detection of associations between qualitative variables. The Mann–Whitney-U or Student’s *t*-test was performed for the comparison between categorical and continuous variables.

The Kaplan–Meier method with a log-rank test was used for mortality analysis and provision of survival estimates. Univariate Cox regression analysis was performed for the detection of potential prognostic factors on the survival. Only significant factors (*p* < 0.05) in the univariate analysis were included in the multivariate analysis. Multivariate Cox regression analysis was performed for the detection of potential independent outcome predictors. All statistical analyses were performed using IBM SPSS Statistics Version 29.0 (SPSS Inc., Chicago, IL, USA).

## 3. Results

### 3.1. Overall Results

#### 3.1.1. Baseline Characteristics

Between January 1995 and December 2020, 980 polytraumatized patients were consecutively enrolled. The clinical characteristics of the patients are listed in Table 1. One-third of patients (30.3%; *n* = 297) were female. The median age of all patients was 39.0 years of age (IQR 29; 17 to 96 years of age), whereas female patients were significantly older than male patients (Mann–Whitney U Test; *p* < 0.001). No significant differences in the length of ICU stay (Mann–Whitney U Test; *p* = 0.259) could be seen, although female patients showed a tendency for longer lengths of hospital stay (Mann–Whitney U Test; *p* = 0.052).

#### 3.1.2. Injury Patterns and Injury Severity

The main mechanism of injury was traffic-related accidents, in 59.8% of patients (*n* = 586), followed by falls from greater height (≥3 m) in 25.5% of patients (*n* = 250), falls from lesser height (≤3 m) (6.1%, *n* = 60), penetrating injuries (2.7%, *n* = 26) and other/unknown causes of injury (5.9%, *n* = 58). Suicidal attempts accounted for 19.4% of patients (*n* = 190) with significantly higher ISS (Mann–Whitney U Test; *p* = 0.003) (Table 1). No sex-related differences regarding the injury pattern could be detected, but female patients presented with significantly higher rates of suicidal attempts (chi-square; 26.1% vs. 16.9%; *p* < 0.001). The median ISS was 34.0 points (IQR 16; 18 to 75 points) with no detected sex-related differences in the injury severity (Mann–Whitney U Test; *p* = 0.537). No sex-related differences regarding injury severity in the body regions of the head (chi-square; *p* = 0.468), thorax (chi-square; *p* = 0.252), abdomen (chi-square; *p* = 0.917) and spine (chi-square; *p* = 0.150) could be seen, but female patients tended to present with higher injury severity in the body region pelvis/extremities compared to male patients (chi-square; AIS > 2 59.7% vs. 52.9%; *p* = 0.051).

#### 3.1.3. Survival

Overall, 287 patients (29.3%) died during the hospital stay, of whom 187 patients (19.1%) died within the acute phase and 100 patients (10.2%) died within the late phase of the trauma. Female patients had significantly higher overall death rates (chi-square; 34.3% vs. 27.1%; *p* = 0.022), although no sex-related differences could be seen with regard to the acute phase and late phase deaths. The main cause of death was trauma/hemorrhage within the acute phase (14.5%, *n* = 142) followed by TBI (9.5%, *n* = 93) and multiple-organ failure (2.6%, *n* = 25), without sex-related differences regarding trauma/hemorrhage (chi-square; *p* = 0.705) or TBI (chi-square; *p* = 0.803).

**Figure 1:**
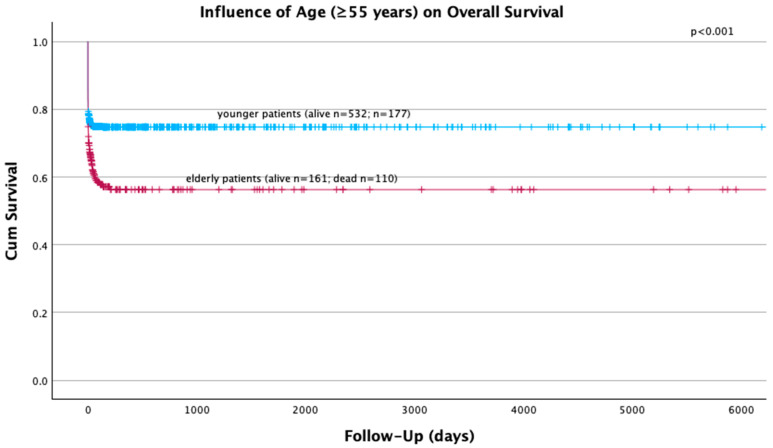
The influence of age (≥55 years of age) on the overall survival of 980 polytraumatized patients.

**Figure 2:**
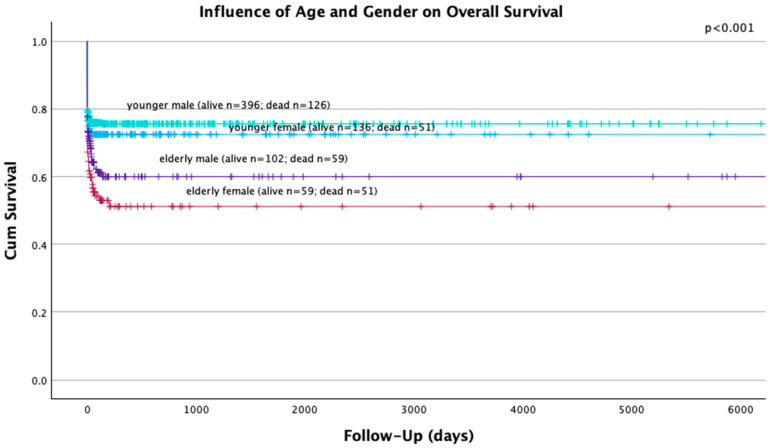
The influence of age and sex on the overall survival of 980 polytraumatized patients.

Univariate analysis showed a significant influence of sex (*p* = 0.027), suicidal attempt (*p* = 0.012), AIS > 2 points in the head (*p* < 0.001), AIS > 2 points in the abdomen (*p* = 0.11) and age ≥ 55 years (Figure 1 and Figure 2) (*p* < 0.001) on overall survival. Multivariate analysis revealed suicidal attempt (HR 1.566; 95% CI 1.150–2.132; *p* = 0.004), AIS > 2 points in the head (HR 1.955, 95% CI 1.475–2.590; *p* < 0.001) and age ≥ 55 years (HR 1.817, 95% CI 1.394–2.368; *p* < 0.001) as independent prognostic factors in the survival of polytraumatized patients.

### 3.2. Sex-Related Differences in Elderly Patients

To overcome any potential beneficial influence of sex hormones in female patients and the negative influence of age on the outcome of polytrauma patients, a sub-analysis of elderly polytrauma patients (≥55 years of age) was performed to detect any sex-related differences.

#### 3.2.1. Baseline Characteristics of Elderly Patients

A total of 271 elderly polytrauma patients were identified; 40.6% (*n* = 110) of them were female. Elderly female patients were significantly older, with a median age of 71 years of age (55 to 96 years) (Mann–Whitney U Test; *p* = 0.014), as seen in Table 2. No differences in the median ISS (median 34 vs. 29 points) could be detected (Mann–Whitney U Test; *p* = 0.093). Elderly female patients had significantly longer lengths of hospital stay compared to male patients (Mann–Whitney U Test; *p* = 0.014).

#### 3.2.2. Injury Patterns and Injury Severity of Elderly Patients

Although no significant sex-related differences in injury patterns could be detected in elderly patients, elderly female patients tended to have higher incidences of suicidal attempts (chi-square; *p* = 0.064). Traffic-related accidents were the most common mechanism of injury in both sex groups, followed by falls from greater and lesser height in nearly the same proportions. Regarding the injury patterns, no sex-related differences regarding injuries to the body regions of the head, thorax, abdomen, pelvis/extremities or spine could be seen (Table 2).

#### 3.2.3. Survival in Elderly Polytrauma Patients

No sex-related differences in elderly polytrauma patients regarding overall death (chi-square; *p* = 0.110), acute-phase death (chi-square; *p* = 0.093) or late-phase death (chi-square; *p* = 0.822) could be detected. Furthermore, no sex-related differences in elderly polytrauma patients regarding the causes of death due to TBI (chi-square; *p* = 0.926) or trauma/hemorrhage (chi-square; *p* = 0.320) could be seen.

## 4. Discussion

There is an ongoing debate on whether sex has an influence on the outcomes of polytraumatized patients [14,15,17,18,19]. Some studies have found a beneficial effect of the female sex on outcomes regarding mortality and the need for ICU admission [14,18], especially in younger female patients, which might be caused by a potential protective effect of sex hormones in these patients [19,21]. Contrarily, other study groups have reported a higher age in female polytraumatized patients with increased LOS [17]. This large retrospective cohort study on polytraumatized patients submitted to a level I trauma center gives new insights into this controversial topic. To overcome the potential beneficial effect of sex hormones on female patients and the potential negative influence of age, a sub-analysis was performed with regard to the elderly population of our polytrauma patients ≥55 years of age.

First, we found that our female patients had a significantly higher median age during the study period, in accordance with previous studies [14,15,17]. Elderly female patients had a significantly longer LOS, suggesting a deficiency in the rehabilitation capability. The increased LOS in elderly female polytraumatized patients might be explained by the higher median age leading to an increased frailty and decreased capability to recover from severe injuries, as suggested in previous studies [17].

Even though no sex-related differences were found in injury patterns, female patients had significantly higher rates of suicidal attempts. Higher rates of female patients were seen in previous studies examining suicidal falls from greater height, with female patients showing more injuries to the pelvis, extremities and lumbar spine [22,23]. Higher rates of severe injuries to the pelvis and extremities in female patients could be observed in our cohort as well and might be explained by higher rates of suicidal attempts in female patients. No sex-related differences regarding injury patterns were found, but with rising age, higher rates of falls from lesser height could be detected in the elderly polytraumatized patient. Still, traffic-related accidents remained the most common cause of trauma in all groups.

Significantly higher overall death rates were found in our cohort of female patients. This finding is in contradiction with previous studies [14,18,24], although recent studies have revealed higher in-hospital mortality rates of female patients within the acute phase [15] and higher overall mortality rates in female patients [15,25]. Contrarily, female patients seem to have a better survival upon extended hospital stay [15]. No sex-related differences regarding the survival of the elderly cohort of our patients could be seen, suggesting that not sex but age is the main prognostic factor for the overall survival (Figure 2). This could additionally be explained by the higher median age of our female patients. The main cause of death remained trauma/hemorrhage within the acute phase and TBI within the late phase, without any sex-related differences.

The age of trauma patients showed a consistent increase over the past decades, with rising incidences of concomitant TBI in elderly trauma patients, as shown by previous studies [26,27]. Consistently, the mortality rates of trauma patients increase with age, especially in the sixth and seventh decade of life [28,29]. Patients with suicidal attempts showed a significantly higher injury severity, with a higher median ISS, which is closely linked to a higher mortality. Merely age, suicidal attempt and severe TBI were found to be the strongest independent prognostic factors in the survival of our overall patient cohort, reflecting similar findings from previous studies [12,30,31]. Still, sex combined with injury pattern, suicidal attempt, age ≥55 years and severe TBI might serve as a prognostic factor in the survival of polytraumatized patients.

This retrospective, single-center analysis has the characteristic limitations of registry data and post hoc analyses. The analyses mostly focused on the mortality of the cohort and there are no data on functional outcome and quality of life parameters available. Although patients were included consecutively and data analysis was performed carefully, there might be an inherent selection bias due to the retrospective design of this study. Further studies focusing on in-hospital recovery, functional outcomes and potential sex-dependent factors underlying frail conditions are needed to explain the discrepant findings of this study.

## 5. Conclusions

Female polytraumatized patients are significantly older and have a prolonged LOS, reflecting potentially higher frailty and a decreased capability to recover from severe injuries. Higher rates of suicidal attempts with slightly higher ISS in the elderly female polytraumatized patient might explain the increased overall mortality rates in female patients. Despite these distinctive sex-related differences, our study suggests a strong sex-independent influence of age and severe TBI on the outcomes of polytraumatized patients.

## Figures and Tables

**Table 1:** Characteristics of all 980 polytraumatized patients (ISS = Injury Severity Score; IQR = Interquartile Range; TBI = Traumatic Brain Injury; MOF = Multiple Organ Failure; AIS = Abbreviated Injury Score).

	All Polytraumatized Patients (*n* = 980)
Female sex	30.3% (*n* = 297)
Male sex	69.7% (*n* = 683)
Age (median)	39 years (IQR 29)
Geriatric patients	27.7% (*n* = 271)
ISS (median)	34 points (IQR 16)
Injury mechanisms	
Traffic-related accident	59.8% (*n* = 586)
Fall from greater height	25.5% (*n* = 250)
Fall from lesser height	6.1% (*n* = 60)
Penetrating injury	2.7% (*n* = 26)
Other/unknown	5.9% (*n* = 58)
Suicidal attempt	19.7% (*n* = 190)
Survival	
Overall death	29.3% (*n* = 287)
Acute-phase death	19.1% (*n* = 187)
Late-phase death	10.2% (*n* = 100)
Causes of death	
Trauma/hemorrhage	14.5% (*n* = 142)
TBI	9.5% (*n* = 93)
MOF	2.6% (*n* = 25)
Injury pattern	
Head (AIS > 2)	59.3% (*n* = 565)
Thorax (AIS > 2)	78.1% (*n* = 742)
Abdomen (AIS > 2)	35.7% (*n* = 337)
Pelvis/extremities (AIS > 2)	55.0% (*n* = 521)
Spine (AIS > 2)	13.1% (*n* = 123)

**Table 2:** Patient characteristics of elderly (≥55 years of age) female and male polytraumatized patients. * Due to the retrospective design of our study, there are some missing values that were not included in the statistical analysis.

	Female (*n* = 110)	Male (*n* = 161)	*p* Value
Median age	67.0 years of age (IQR 17)		
Age	70.18 ± 10.33	67.11 ± 10.03	0.008 *
Geriatric patients			
Median ISS	33 points (IQR 16)		
ISS	35.70 ± 14.52	33.51 ± 14.26	0.110
Injury mechanism			
Traffic-related accident	62.8% (*n* = 68)	58.4% (*n* = 94)	0.899
Fall from greater height	15.5% (*n* = 17)	18.0% (*n* = 29)	
Fall from lesser height	16.4% (*n* = 18)	14.9% (*n* = 24)	
Penetrating injury	0.9% (*n* = 1)	1.9% (*n* = 3)	
Other/unknown	5.5% (*n* = 6)	6.8% (*n* = 11)	
Suicidal attempt	11.9% (*n* = 13)	5.6% (*n* = 9)	0.064
Survival			
Overall death	46.4% (*n* = 51)	36.6% (*n* = 59)	0.110
Acute-phase death	27.3% (*n* = 30)	18.6% (*n* = 30)	0.093
Late-phase death	19.1% (*n* = 21)	18.0% (*n* = 29)	0.822
Causes of death			
Trauma/hemorrhage	20.0% (*n* = 22)	12.4% (*n* = 20)	0.390
TBI	14.5% (*n* = 16)	11.8% (*n* = 19)	
MOF	4.5% (*n* = 5)	6.2% (*n* = 10)	
Injury pattern			
Head (AIS > 2)	69.8% (*n* = 74)	62.7% (*n* = 99)	0.231
Thorax (AIS > 2)	75.7% (*n* = 81)	75.3% (*n* = 119)	0.943
Abdomen (AIS > 2)	24.3% (*n* = 25)	26.1% (*n* = 40)	0.736
Pelvis/extremities (AIS > 2)	57.0% (*n* = 61)	49.7% (*n* = 77)	0.243
Spine (AIS > 2)	10.8% (*n* = 11)	10.7% (*n* = 16)	0.976

## Data Availability

The data used and analyzed in this study are available from the corresponding author solely upon reason.
